# Unleashing “Civilian Power”: A New American Diplomacy through Neglected Tropical Disease Control, Elimination, Research, and Development

**DOI:** 10.1371/journal.pntd.0001134

**Published:** 2011-06-28

**Authors:** Peter J. Hotez

**Affiliations:** 1 Sabin Vaccine Institute – Texas Children's Hospital – Baylor College of Medicine Center for Vaccine Development, Houston, Texas, United States of America; 2 Departments of Pediatrics and Molecular Virology & Microbiology, and National School of Tropical Medicine, Baylor College of Medicine, Houston Texas, United States of America

## 

In a November/December 2010 article published in the influential journal *Foreign Affairs*, United States Secretary of State Hilary Rodham Clinton articulated a new vision for American diplomacy and development through the strengthening of what she terms “civilian power” [1]. Stated briefly, the new doctrine calls for the creation of a new cadre of civilian experts who could jointly pursue diplomacy and international development for purposes of solving global challenges related to health, agriculture and food insecurity, environmental degradation, drugs and organized crime, energy, and climate change [1]. Through joint programs between the US Department of State and its United States Agency for International Development (USAID) and the additional hiring of more than 1,000 Foreign and Civil Service Officers in each organization, and reflecting the results of a new Quadrennial Diplomacy and Development Review, in the coming years the State Department and USAID would now work more closely to enhance global development in the context of diplomacy. The world's conflict and post-conflict zones and fragile states would represent key targets for international development efforts [1]. In this new doctrine, Secretary Clinton proposes that together the State Department and USAID would establish a premier global civilian service for responding to complex diplomatic and development challenges [1]. Achieving such an ambitious goal would also mandate that USAID look beyond its walls to embrace business, philanthropist, and citizen groups, with dual emphases on partnering with some of the large emerging market economies (EMEs), i.e., China, India, Indonesia, Nigeria, Pakistan, Russia, and South Africa, for joint problem solving, and harnessing selected technologies, such as rapidly expanding cell phone access, for establishing a sustainable and lasting impact [1].

The control and elimination of the world's major neglected tropical diseases (NTDs) represents a substantive global health and economic challenge, yet one that with a well-coordinated assault in the mode as proposed by Secretary Clinton could become the first major victory for civilian power. The NTDs are the most common infections of the world's poor, and almost all of the “bottom billion,” i.e., the 1.4 billion people, who live on no money, are infected with one or more NTD [2], [3]. In addition to causing a level of global disability that compares with that of HIV/AIDS or malaria, NTDs actually cause poverty through their ability to impair child development, pregnancy outcomes, and agricultural productivity and food security [3]. These diseases also destabilize communities and promote civil and international conflicts [4].

There are a number of reasons why taking on the NTDs would be a worthy early first test for civilian power. It is now possible to control or in some cases eliminate one or more of the seven most common NTDs, i.e., ascariasis, hookworm infection, trichuriasis, schistosomiasis, lymphatic filariasis, onchocerciasis, and trachoma, through mass drug administration using a “rapid impact package” of drugs that are either donated by the major pharmaceutical companies or available as low cost generics and administered as a part of national control programs [2], [3], [5]. Mass drug administration for NTDs through rapid impact is one of the lowest cost and cost-efficient mechanisms for both improving maternal child health [6] and lifting the bottom billion out of poverty [3]. The United States government (USG), through its Global Health Initiative (GHI) and USAID's NTD Program, is already providing financial support for 12 national NTD programs, more than all other countries combined, with additional programs scheduled in the coming years [7].

With assistance from the Department of State, the USAID NTD Program can now reach out to the major EMEs listed above in order to join forces on global NTD control efforts. A USAID–EMEs link makes sense on two fronts: First, it has already been noted that Nigeria has the largest number people infected with the seven NTDs among the sub-Saharan African countries, while South Africa suffers from high rates of some of the soil-transmitted helminth infections and schistosomiasis [8]. Similarly, China, India, Indonesia, and Pakistan have the largest number of ascariasis, trichuriasis, hookworm, lymphatic filariasis, and trachoma cases, as well as other NTDs such as leprosy in Asia [9], [10]. Working jointly with experts from USAID and other branches of the USG and GHI, a focused effort on NTDs in the EMEs could ultimately double the total number of at-risk populations receiving rapid impact packages. In addition, China invests billions of dollars in sub-Saharan Africa annually and could also make substantial contributions to NTD control on the African continent, possibly doubling yet again the USAID contribution [11]. In time, USAID and the EMEs could combine to target most of the people at risk in Africa and Asia from NTDs with rapid impact packages, thereby helping to affect lymphatic filariasis, onchocerciasis, and trachoma eliminations and advancing soil-transmitted helminth infection and schistosomiasis control in many of these countries.

Strengthening fragile and failing states is a key element of Secretary Clinton's doctrine for civilian power [1]. Some of the highest rates of NTDs occur in conflict and post-conflict countries [4], and in fragile nations such as Mali, Mauritania, Niger, Pakistan, Somalia, and Yemen where, according to Secretary Clinton [1], Al Qaeda operates [12]. Because the NTDs destabilize these communities and also represent important impediments to human rights [4], [13], NTD control should comprise an essential element of civilian power for the reinvention of American diplomacy and development. The business and philanthropic communities can also be engaged in global NTD efforts through the establishment of a new private fund (End Neglected Diseases, END) established jointly by Legatum and Geneva Global [14], and supported by the non-profit Global Network for Neglected Tropical Diseases, while simultaneously continuing to work jointly with the major pharmaceutical companies for ensuring ongoing commitments for existing NTD drug donations [5], as well as expanding commitments to provide urgently needed essential medicines such as praziquantel [15].

USAID could also engage EME and private partners in international research and development efforts to generate and produce new NTD control tools such as drugs, vaccines, and diagnostics [13]. Such products are sometimes referred to as antipoverty technologies [13], [16]. Currently neither USAID nor GHI provide significant support for NTD antipoverty technologies, but even a 1%–2% allocation of the total GHI budget could mobilize an estimated US$100–200 million annually for this purpose [16]. The EME countries, but especially Brazil and India, are actively pursuing the development and testing of new NTD products [17]. The USG and major US research universities are failing to take advantage of an incredible opportunity to expand joint research and development activities with the EMEs, beyond the current product development partnership activities and the Tropical Medicine Research Centers supported by the National Institutes of Health [16], by not incorporating NTD product development into a larger program of science and technology diplomacy [18]. Vaccine diplomacy also represents an exciting opportunity to jointly pursue antipoverty vaccine development with some of the large middle-income countries that comprise the Organisation of the Islamic Conference (OIC), i.e., technologically proficient nations such as Indonesia, Iran, and Pakistan [18]. Such activities could link with the major multinational pharmaceutical companies and would be consistent with Secretary Clinton's calls for expanded use of technology for diplomacy and development [1]. An expanded USG initiative in this context could also lead to greater global investment in NTD research just as the 2009 Group of Eight (G8) meeting in Italy led the US to secure a US$20 billion international commitment for food security [1].

A global assault on NTDs both through expanded deployment of rapid impact packages and research and development for new drugs, vaccines, and diagnostics would represent a modern day Marshall Plan for the world's developing countries. Conducted jointly with the EMEs and leading OIC countries, the global NTD assault would maximize civilian power in order to achieve the diplomatic and development goals of the USG.

## References

[1] Clinton HR (2010). Leading through civilian power: redefining American diplomacy and development.. Foreign Affairs.

[2] Hotez PJ, Molyneux DH, Fenwick A, Kumaresan J, Ehrlich Sachs S (2007). Control of neglected tropical diseases.. N Engl J Med.

[3] Hotez PJ, Fenwick A, Savioli L, Molyneux DH (2009). Rescuing the bottom billion through control of neglected tropical diseases.. Lancet.

[4] Hotez PJ, Thompson TG (2009). Waging peace through neglected tropical disease control: a U.S. foreign policy for the bottom billion.. PLoS Negl Trop Dis.

[5] Hotez PJ (2009). Mass drug administration and the integrated control of the world's high prevalence neglected tropical diseases.. Clin Pharmacol Therap.

[6] Hotez PJ (2009). Empowering women and improving female reproductive health through control of the neglected tropical diseases.. PLoS Negl Trop Dis.

[7] http://www.neglecteddiseases.gov, accessed November 21, 2010

[8] Hotez PJ, Kamath A (2009). Neglected tropical diseases in sub-Saharan Africa: review of their prevalence, distribution and disease burden.. PLoS Negl Trop Dis.

[9] Hotez PJ, Ehrenberg J (2010). Escalating the global fight against neglected tropical diseases through interventions in the Asia Pacific Region.. Adv Parasitol.

[10] Hotez PJ (2010). Nuclear weapons and neglected diseases: the “ten thousand to one gap.”. PLoS Negl Trop.

[11] Hotez PJ (2010). Neglected tropical disease control in the “Post-American world”.. PLoS Negl Trop Dis.

[12] Hotez PJ (2009). The neglected tropical diseases and their devastating health and economic impact on the member nations of the organization of the Islamic Conference.. PLoS Negl Trop Dis.

[13] Hotez PJ, Pecoul B (2010). “Manifesto” for the control and elimination of the neglected tropical diseases.. PLoS Negl Trop Dis.

[14] Hotez PJ (2010). A plan to defeat neglected tropical diseases.. Sci Am.

[15] Hotez PJ, Engels D, Fenwick A, Savioli L (2010). Africa is desperate for praziquantel.. Lancet.

[16] Hotez PJ (2011). New antipoverty vaccines and drugs: a research agenda for the U.S. President's Global Health Initiative (GHI).. PLoS Negl Trop Dis.

[17] Morel CM, Acharya T, Broun D, Dangi A, Elias C (2005). Health innovation: the neglected capacity of developing countries to address neglected diseases.. Science.

[18] Hotez PJ (2010). Peace through vaccine diplomacy.. Science.

